# Update on the Epimed Monitor Adult ICU Database: 15 years of its use in national registries, quality improvement initiatives and clinical research

**DOI:** 10.62675/2965-2774.20240150-en

**Published:** 2024-08-15

**Authors:** Marcio Soares, Lunna Perdigão Borges, Leonardo dos Santos Lourenco Bastos, Fernando Godinho Zampieri, Gabriel Alves Miranda, Pedro Kurtz, Suzana Margareth Lobo, Lucas Rodrigo Garcia de Mello, Gastón Burghi, Ederlon Rezende, Otávio Tavares Ranzani, Jorge Ibrain Figueira Salluh

**Affiliations:** 1 Instituto D’Or de Pesquisa e Ensino Rio de Janeiro RJ Brazil Instituto D’Or de Pesquisa e Ensino - Rio de Janeiro (RJ), Brazil.; 2 Epimed Solutions Rio de Janeiro RJ Brazil Epimed Solutions - Rio de Janeiro (RJ), Brazil.; 3 Pontifícia Universidade Católica do Rio de Janeiro Department of Industrial Engineering Rio de Janeiro RJ Brazil Department of Industrial Engineering, Pontifícia Universidade Católica do Rio de Janeiro - Rio de Janeiro (RJ), Brazil.; 4 University of Alberta Faculty of Medicine and Dentistry Edmonton Canada Faculty of Medicine and Dentistry, University of Alberta - Edmonton, Canada.; 5 Faculdade de Medicina de São José do Rio Preto Hospital de Base Intensive Care Division São José do Rio Preto SP Brazil Intensive Care Division. Hospital de Base, Faculdade de Medicina de São José do Rio Preto - São José do Rio Preto (SP), Brazil.; 6 Hospital Maciel Intensive Care Unit Montevideo Uruguay Intensive Care Unit, Hospital Maciel - Montevideo, Uruguay.; 7 Hospital do Servidor Público Estadual "Francisco Morato de Oliveira" Intensive Care Unit São Paulo SP Brazil Intensive Care Unit, Hospital do Servidor Público Estadual "Francisco Morato de Oliveira" - São Paulo (SP), Brazil.; 8 Barcelona Institute for Global Health Barcelona Spain Barcelona Institute for Global Health (ISGlobal) - Barcelona, Spain.

**Keywords:** Databases, factual, Registries, Quality improvement, Hospital mortality, Critical care, Intensive care units

## Abstract

In recent decades, several databases of critically ill patients have become available in both low-, middle-, and high-income countries from all continents. These databases are also rich sources of data for the surveillance of emerging diseases, intensive care unit performance evaluation and benchmarking, quality improvement projects and clinical research. The Epimed Monitor database is turning 15 years old in 2024 and has become one of the largest of these databases. In recent years, there has been rapid geographical expansion, an increase in the number of participating intensive care units and hospitals, and the addition of several new variables and scores, allowing a more complete characterization of patients to facilitate multicenter clinical studies. As of December 2023, the database was being used regularly for 23,852 beds in 1,723 intensive care units and 763 hospitals from ten countries, totaling more than 5.6 million admissions. In addition, critical care societies have adopted the system and its database to establish national registries and international collaborations. In the present review, we provide an updated description of the database; report experiences of its use in critical care for quality improvement initiatives, national registries and clinical research; and explore other potential future perspectives and developments.

## INTRODUCTION

In recent decades, the growing interest in clinical research, epidemiology, quality improvement and intensive care unit (ICU) performance evaluation coupled with the increasing adoption of health care informatics has resulted in the establishment of large databases of critically ill patients internationally. Currently, some contemporaneous large datasets comprising granular data on ICU admission, which are obtained from electronic medical records fully dedicated to clinical research, provide open access to data for investigators.^( 1 )^ In addition, large regional and national databases gathering data on case mix, resource use and outcomes of consecutive ICU patients, which are referred to as ICU registries, have been developed and implemented worldwide.^( 2 )^ These initiatives both in low- and in middle- and high-income countries from all continents are rich sources of data for the surveillance of emerging diseases, ICU performance evaluation and benchmarking, quality improvement projects and clinical research (ranging from observational studies to randomized clinical trials [RCT]-embedded trials).

The Epimed Monitor database, which is a cloud-based platform for clinical performance management and benchmarking for ICUs, was created 15 years ago.^( 3 )^ Since 2009, the database has grown in the number of ICUs and admissions and has expanded to other countries beyond Brazil, becoming one of the largest critical care databases worldwide. The initial purpose of the database was to evaluate ICU performance and monitor quality improvement initiatives by ICU and hospital administrators. In 2017, a study published in this journal described the potential of the platform and database contents for critical care research in Brazil.^( 3 )^ Over the years, critical care societies have also adopted the system and its database to establish national registries and international collaborations, such as the Brazilian Intensive Care Units Registry (" *UTIs Brasileiras* ").^( 4 , 5 )^ In addition, the database was enriched with several new variables and scores following scientific advances in both technical and clinical research. Therefore, the aims of the present review are to update the description of the database; to report experiences of its use in critical care in national registries, quality improvement initiatives and clinical research; and to explore other potential future perspectives and developments. Although there are versions of the system for pediatric and neonatal ICUs, we focus here on the adult ICU database only.

### Database description

#### Participation in the Epimed Monitor database

Participation in the Epimed Monitor database in all countries is voluntary and regulated by a commercial contract with an information technology company (Epimed Solutions^®^, Rio de Janeiro, Brazil), which is responsible for system development, updating, security, data privacy and protection, backup, and support to all users. The majority of ICUs use the complete version of the system, but a few ICUs use a standard free version available in the national registries. The system is available in Dutch, English, French, German, Portuguese, and Spanish. Words and terms may be adapted to meet local cultural specificities (e.g., Portuguese in Brazil or Portugal), as needed while keeping the same internal codes.

#### Data entry, processing, and quality control

Data are entered into a structured and hierarchical electronic case report form (eCRF), which has a basic compulsory data frame, combining integration with the hospital's electronic (medical and/or administrative) records (HER) and manual data entry depending on the hospitals’ information technology infrastructure, which can range from full manual data entry to full HER integration. In most ICUs, administrative (demographics, admission, and discharge information) and laboratory data are integrated, and a dedicated case manager (usually nurses) is responsible for entering clinical data for every consecutive patient into the database. The company provides case managers with initial training followed by continuing educational programs and periodic updates and feedback. Online/live training also occurs, with regular (at least bimonthly) face-to-face meetings with the users, in which, for instance, data quality and completeness can be rechecked with users as needed. In general, admissions are entered prospectively. Otherwise, charts are reviewed, avoiding data loss, especially in patients admitted to the ICU on weekends or patients who die within 24 hours after admission. For specific eCRF sections [e.g., infection-related data, adverse events, Nurse Activities Score (NAS) and checklists], other multidisciplinary team members may be involved in data entry. In particular, few customizations are performed to comply with local regulations, depending on the country.

Each admission is assigned a unique sequence identifier, which follows the order of the whole database and not each unit, hospital, or country. In the case of readmissions, even during the same hospitalization, a new unique identifier number is also assigned.

Although there are no regular external audits, the database is structured to have active controls to guarantee data quality and consistency. There are no free text fields, and all the variables are structured with internal linked codes. To minimize processing errors, which encompass coding and data entry steps, the definitions/labels of each variable not only are clearly stated in the eCRF but are also available in a PDF sheet that is easily accessible on the online platform. To address possible errors, the system provides internal validation routines during the data entry process ("interactive checking"). Inconsistent date ranges are not allowed. Conditional filling is also present for some specific variables (e.g., diagnoses, pathogens, and antibiotics). For physiological and laboratory data, values beyond the usual range for the variable are highlighted for review, and implausible values cannot be recorded. Intensive care unit coordinators and case managers can monitor incomplete cases and evaluate the proportion of missing values or the incompleteness of specific variables over a period. In addition, offline checks can occur at random, depending on the demand for each unit and for database updating and improvement.

#### Electronic case report form structure

The eCRF is hierarchically structured into specific datasheets for both time-independent and time-dependent variables ( Table 1S - Supplementary Material ). Each data point entered in the database is followed by a calendar date to keep the log.

Demographic and administrative data include unique identifiers, age, sex, whether the readmission occurred during the same hospitalization (and whether the readmission occurred within 24, 48 or 72 hours of ICU discharge), weight, height, bed number, source of admission, hospital and ICU admission and discharge dates and time, and destination after the ICU and hospital discharge. Comorbidities include all comorbidities from the Charlson Comorbidity Index (CCI),^( 6 )^ the Simplified Acute Physiology Score (SAPS) 3,^( 7 )^ the Modified Frailty Index (MFI),^( 8 , 9 )^ the Acute Physiology and Chronic Health Evaluation (APACHE) II,^( 10 )^ and additional comorbidities that may be useful for risk assessment and stratification on specific conditions (e.g., stroke, coronary artery disease). Data concerning chronic health status in the week prior to hospital admission were adapted from the Eastern Cooperative Oncology Group (ECOG) performance status.^( 11 , 12 )^

Admissions are classified as medical, elective surgery or emergency/urgent surgery on the basis of the initial diagnosis classification. A list of more than 1,500 prespecified medical and surgical diagnoses is available and organized in several domains ( Table 2S - Supplementary Material ). Secondary diagnoses at admission and during the stay can also be recorded. A codification based on the International Classification of Diseases - Tenth Revision (ICD-10) is available.

Table 1 shows invasive organ support and acute complications at admission (± 1 hour) and within 24 hours of ICU admission considered in the database. Laboratory and physiological data at admission and during the first 24 hours are also collected, representing those needed to calculate the severity of illness scores accordingly. Invasive support and interventions during the ICU stay include invasive monitoring and interventions (minimally invasive hemodynamic monitoring, central venous (CVC), bladder, Swan-Ganz, and arterial catheters), neurological support and monitoring (intracranial pressure, monitoring of tissue oxygen pressure], jugular venous oxygen saturation, cerebral microdialysis and external ventricular drainage), ventilatory support (noninvasive ventilation, mechanical ventilation [MV], MV duration, tracheostomy, high-flow nasal cannula and inhaled nitric oxide), cardiovascular support (transvenous pacemaker, intra-aortic balloon pump catheter, vasoactive drugs, and extracorporeal membrane oxygenation (ECMO)), renal replacement therapy (RRT), blood transfusions, thrombolytic agents, parenteral nutrition, therapeutic hypothermia and plasmapheresis.

Intensive care unit prioritization frameworks recommended by the Society of Critical Care Medicine (SCCM)^( 13 )^ and by the *Conselho Federal de Medicina* (CFM)/ *Associação de Medicina Intensiva Brasileira* (AMIB) (Resolução CFM 2.156/2016)^( 14 )^ and decisions to prioritize palliative care and end-of-life decisions were updated from the last description of the database.

Several ICUs also record daily checklists for adherence to best evidence practices (sedation, invasive device care, MV, ulcer pressure prevention, sepsis bundles, and bundles for prevention of hospital-acquired infections).

#### Scoring systems

Several scoring systems are available in the database for different purposes and domains ( Table 3S - Supplementary Material ). The scores calculated from the compulsory data are SAPS 3, CCI and MFI, as described previously. The Sequential Organ Failure Assessment (SOFA) score can also be estimated on the first day of the ICU and daily into dynamic datasheets or mobile apps. Several ICUs assess the nurse workload via the NAS.^( 15 )^ In addition, a few ICUs also use APACHE II and SAPS 2 scores.

Recently, Epimed Solutions^®^ developed a series of proprietary scores, the Epimed Prediction Model (EPM), which uses machine learning modeling in contemporary datasets of more than one million patients. There are EPMs for estimating the risk for hospital mortality, the ICU length of stay (LOS), the risk for prolonged LOS (longer than the 90^th^ percentile of LOS for each given diagnosis) and the risk for ICU readmission within 48 hours after discharge. The performance of all scores is periodically reassessed for the need for updates as appropriate.^( 16 )^

#### Quality indicators and performance evaluation

Monitoring of quality indicators and assessments of ICU performance and efficiency are the main purposes for use of the system by ICUs and hospitals. Core quality indicators are those recommended by local national societies, including *Agência Nacional de Vigilância Sanitária* (ANVISA),^( 17 , 18 )^ Brazil, and the European Society of Intensive Care Medicine (ESICM) task force.^( 19 )^ The quality indicators in the database include ICU and hospital mortality rates; standardized mortality ratios (SMRs) using severity-of-illness scores; early unplanned ICU readmissions (within 24, 48 and 72 hours of discharge); ICU and hospital LOS; invasive device use rates (MV, CVC and bladder catheter); incidence rates of health care-associated infections (ventilator-associated pneumonia, central line-associated bloodstream infection, and catheter-associated urinary tract infection); and qualitative and quantitative evaluations of nurse workload and adherence to bundles to prevent health care-associated infections. The Epimed Monitor database provides surveillance for incidents and adverse events, such as transfusion-related incidents and complications, drug-induced adverse events, unintended extubation, and pressure ulcers, among other situations.

In addition to SMRs used to evaluate clinical performance, two other measures are provided to measure the efficiency of resource use. The standardized resource use ratio (SRU) estimates the average observed-to-expected ratio of resources (based on ICU LOS) used per surviving patient in a specific ICU adjusted for the SAPS 3 or EPM mortality.^( 20 )^ The intensive care unit efficiency can be analyzed by combining the SMR and the SRU in an efficiency matrix.^( 21 , 22 )^ The system also provides the standardized length of stay (SLOS), which is the ratio between the observed and the predicted ICU LOS on the basis of the EPM length of stay.

### Participating intensive care units and patient characteristics

#### Geographic distribution and growth of the database

**Figure 1:**
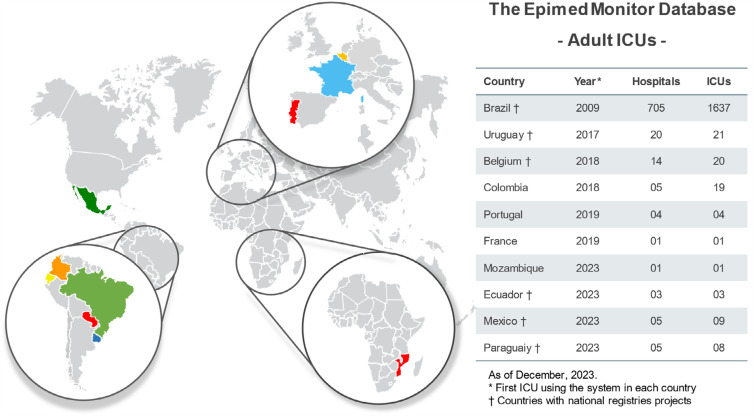
Number of hospitals and intensive care units in countries using the Epimed Monitor Adult ICU Database with the respective starting years.

**Figure 2:**
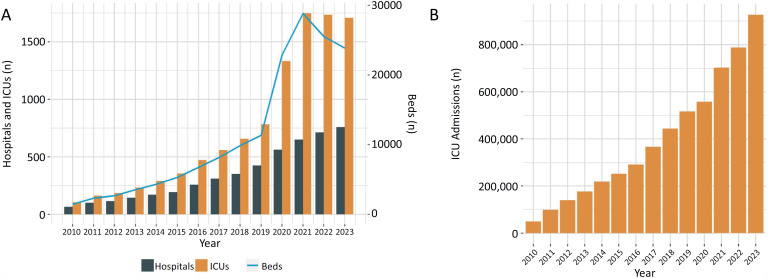
Yearly (2010 - 2023) trends in (A) the number of hospitals, intensive care units and intensive care unit beds and (B) the total number of intensive care unit admissions.

As of December 2023, the Epimed Monitor Adult ICU System was used regularly in 23,852 beds in 1,723 ICUs and 763 hospitals from ten countries ( Figure 1 ). In Brazil, the system has been adopted by ICUs, which represent approximately 50% of adult critical care beds in 25 out of the 27 Brazilian states.^( 4 )^ Figures 2A and 2B show the continuous growth of the database since 2010. In 2020 and 2021, there was a steep increase in the number of beds and ICUs needed to meet the demands of coronavirus disease 2019 [COVID-19], with a subsequent decrease as the pandemic was progressively controlled ( Figure 2A ). The number of ICU admissions has also increased progressively, surpassing more than 5.6 million in total, and it is expected to exceed one million admissions per year in 2024 ( Figure 2B ).

ICU - intensive care unit.

ICU - intensive care unit.

#### Admission diagnosis, organ support use and outcomes

**Figure 3:**
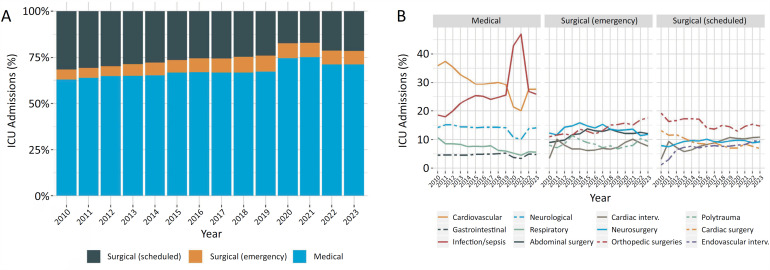
Yearly (2010 - 2023) trends in (A) the proportion of patients according to the admission type and (B) the five most frequent diagnosis categories within each admission type.

Figures 3A and 3B illustrate the profile of ICU admission diagnoses. On average, two-thirds were medical admissions, which, as expected, increased during the COVID-19 pandemic in parallel with a decrease in the proportion of scheduled surgical patients. Figure 3B shows the five most frequent diagnosis categories according to the admission type.

ICU - intensive care unit.

**Figure 4:**
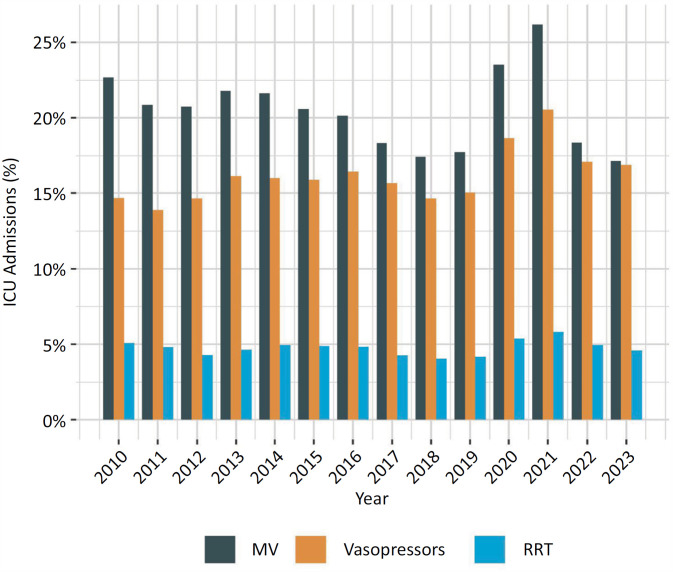
Yearly (2010 - 2023) trends in the frequency of mechanical ventilation, vasopressor use and renal replacement therapy.

**Figure 5:**
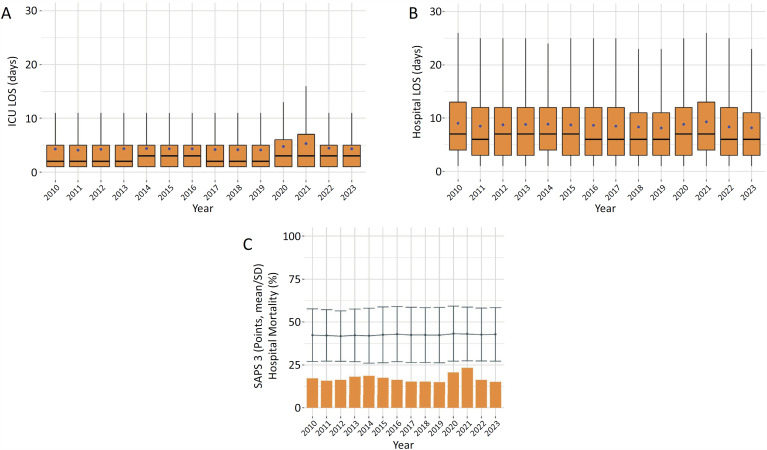
Yearly (2010 - 2023) trends in (A) intensive care unit and (B) hospital lengths of stay (boxplot; the blue dots represent the mean value); (C) mean Simplified Acute Physiology Score 3 (with respective 95% confidence intervals) and hospital mortality rate.

Figures 4 and 5 depict the trends of the main invasive support use and outcomes over time, respectively. As expected, the results from 2020 - 2022 were impacted by the disproportionate number of severe patients with COVID-19 admitted to ICUs during these years. However, in 2023, these data were again more comparable to the prepandemic findings. The most frequent organ support interventions over the years are shown in figure 4 . From 2010 to 2019, there was a trend toward a decrease in the proportion of patients requiring MV, which may be explained in part by the broader use of noninvasive ventilation methods more recently. On the other hand, the frequency of vasopressor and RRT use has remained relatively stable over the years. The same pattern of invasive support use trends was observed for patient outcomes. The ICU LOS remained stable, but there was a modest decrease in the hospital LOS across the prepandemic period ( Figures 5A and 5B ). Similarly, there was a small decrease in hospital mortality, which was not explained by changes in the SAPS 3 score ( Figure 5C ). In addition, during the COVID-19 pandemic, SAPS 3 did not change in relation to the nonpandemic years, reinforcing the inaccuracy of its performance in COVID-19 patients.^( 23 )^

ICU - intensive care unit; MV - mechanical ventilation; RRT - renal replacement therapy.

ICU - intensive care unit; LOS - length of stay; SAPS 3 - Simplified Acute Physiology Score 3; SD - standard deviation.

### National registries adopting the database

The Brazilian ICU Registry (" *UTIs Brasileiras* ") was the first national registry supported by the Epimed Monitor database by AMIB.^( 4 , 5 )^ Starting in 2010, the " *UTIs Brasileiras* " now encompass approximately 50% of all ICU beds in Brazil. It was followed by the Uruguayan registry in 2016^( 24 )^ and Belgium in 2017.^( 25 )^ By the end of 2023, the database was used in ten countries, six of which have adopted it in the context of establishing national registries ( Figure 1 ). Currently, under the support of the Pan American and Iberian Federation of Critical Medicine and Intensive Care (FEPIMCTI), a project named "UCIs Ibero-Americanas" was started to engage countries in the region to start their own national registries sharing the same platform.^( 26 )^ In addition to Brazil and Uruguay, the following countries have agreed to participate: Mexico, Panama, Colombia, Ecuador, Chile, Peru, Paraguay and countries represented by Central America and the Caribbean Consortium of Critical Care.

### Use in quality improvement initiatives

In addition to national-level initiatives through ICU registries, the system and database are also used to assist and monitor quality improvement initiatives in ICUs and hospitals. A large network of hospitals employed the system to monitor the implementation of process measures associated with audits and feedback to improve the care and outcomes of patients with sepsis and minimize sedation.^( 27 , 28 )^

### Clinical research

In a nonsystematic search of the PubMed database, we identified 44 articles on critically ill adult patients whose data were partially or completely retrieved from the Epimed Monitor ( Table 4S - Supplementary Material ). There were 36 (82%) multicenter studies. Most studies included ICUs from Brazil only (n = 36, 82%), and eight were performed in ICUs from other countries, including four multinational studies. The median number of patients per study was 13,301 (ranging from 100 to more than 1,300,000). The main topics of interest were ICU organization and performance (n=10, 23%), COVID-19 (n = 8, 18%), ICU case mix and outcomes (n = 6, 14%), critically ill patients with cancer (n = 6, 14%), infection/sepsis (n = 6, 14%), and neurocritical care (n = 4, 9%), among others. Notably, the ORCHESTRA (Organizational CHaractEriSTics in cRitical cAre) study, which aims to investigate the associations among patient characteristics, ICU organizational aspects, patient outcomes and performance and efficiency in ICUs, is the largest in number of publications.^( 9 , 12 , 16 , 22 , 29 - 40 )^ That study has been conducted over a ten-year period and includes data from more than 600,000 patients and 250 ICUs from Brazil and Uruguay.

### Future perspectives

The growth of the Epimed Monitor Adult ICU Database in terms of countries, hospitals, ICUs, and patients will create opportunities to leverage collaborative multicenter and multinational studies with harmonized, high-quality standardized data. Moreover, it can mitigate the effort and burden of conducting clinical trials, since a comprehensive clinical characterization of patients, support use and outcomes has already been routinely collected prospectively, as has been occurring in other countries, such as Australia, New Zealand, and the United Kingdom. This database may allow the identification of centers with targeted patient populations for those studies; support realistic sample size, power and recruitment estimations for clinical trials; and facilitate registry-embedded trials.^( 40 )^ Investigators can conduct studies to validate and test existing scores and measures regularly used in critical care patients and check for the eventual need for updates and local customizations via contemporary data.^( 16 , 23 , 36 )^ In the future, this database may play a role in tracking evolution and ICU bed occupation and providing almost real-time information on patients during pandemics, such as those that have occurred with COVID-19.^( 5 )^ Finally, several new perspectives will be opened with the availability of new modeling algorithms using machine learning and other "artificial intelligence" techniques.

## CONCLUSION

The Epimed Monitor Adult ICU Database is one of the largest critical care databases worldwide. In recent years, there has been rapid geographical expansion and an increase in the number of participating intensive care units and hospitals, with several new variables and scores added, allowing a more complete characterization of patients to foster multicenter clinical studies. An increasing number of countries are adopting databases to promote national intensive care unit registries, monitor intensive care unit performance and efficiency, and support quality improvement initiatives.

## SUPPLEMENTARY MATERIAL


