# Supplementary material for: Update on the Epimed Monitor Adult ICU Database: 15 years of its use in national registries, quality improvement initiatives and clinical research
Source: Crit Care Sci. 2024 Aug 15;36:e20240150en. doi: 10.62675/2965-2774.20240150-en (PMC11463981; doi:10.62675/2965-2774.20240150-en)
Supplement: Supplementary file 1 [file 2965-2774-ccsci-36-e20240150en-suppl01.pdf]

Critical Care Science
https://doi.org/10.62675/2965-2774.20240150-en

SUPPLEMENTARY MATERIAL

Update of the Epimed Monitor Adult ICU Database: 15 years of its use in national registries, quality improvement initiatives and clinical research

Marcio Soares[1], Lunna Perdigão Borges[2], Leonardo dos Santos Lourenco Bastos[3], Fernando Godinho Zampieri[4], Gabriel Alves Miranda[2], Pedro Kurtz[1], Suzana Margareth Lobo[5], Lucas Rodrigo Garcia de Mello[2], Gastón Burghi[6], Ederlon Rezende[7], Otávio Tavares Ranzani[8], Jorge Ibrain Figueira Salluh[1]

Table 1S - Electronic case report form datasheets and domains

Data domains	Variables
Demographics*	Age, sex
Unit admission*	ICU and hospital admission date, hospital and ICU readmission status, source of admission, weight, height, and BMI at admission
Functional capacity and comorbidities*	Pre-hospital functional capacity and comorbidities. Please see text for details.
Diagnosis*	ICU admission diagnosis and secondary diagnoses during the ICU stay
SAPS 3 admission diagnosis*	ICU admissions diagnosis and conditions encompassed by the SAPS 3 score[7]
Support and complications on Day 1*	Specific acute complications (intracranial mass effect, acute respiratory failure, neutropenia), cardiac arrhythmias, cardiac arrest, invasive support (noninvasive ventilation, mechanical ventilation, vasopressors and MV) at ICU admission (± 1 hour) and in the first 24 hours
Physiological and laboratory data (1 hour)*	Blood pressure, respiratory and heart rates, temperature, Glasgow coma scale, leukocyte and platelets counts, creatinine, urea, bilirubin, blood gas analysis, lactate and FiO_2
Physiological and laboratory data (24 hours) (optional)	Same as above with the addition of urine output, hematocrit, hemoglobin, sodium, potassium, NRI, albumin, C-reactive protein
Invasive devices*	Invasive devices and treatments with start and end dates: MV, intravascular catheters (including intra-aortic ballon pump, transvenous pacemakers and others), ventricular shunts, dialysis, ECMO, bladder catheter
Support and interventions in the ICU	Please refer to text
Infections (optional)	Infection-related data: infection site, source and proof, pathogens and antibiotics
ICU prioritization and palliative care (optional)	SCCM or CFM/AMIB ICU prioritization framework,[13,14] prioritization of palliative care and end-of-life decisions
Adverse events (optional)	ICU-acquired incidents and adverse events
ICU outcomes*	ICU discharge date, vital status, destination after discharge, postdischarge ICU readmission (within 24, 48 and 72 hours)
Hospital outcomes*	Hospital discharge date, vital status, destination after discharge, postdischarge hospital readmission
Scores	Please refer to text and table 3S

ICU - intensive care unit; BMI - body mass index; SAPS- Simplified Acute Physiology Score; MV - mechanical ventilation; ECMO - extracorporeal membrane oxygenation; SCCM - Society of Critical Care Medicine; CFM - *Conselho Federal de Medicina*; AMIB - *Associação de Medicina Intensiva Brasileira*. * Mandatory data.

This is an open access article under the CC BY license (https://creativecommons.org/licenses/by/4.0/).

Table 2S - Medical and surgical diagnoses categories

Medical	Surgical
Bone, joint and soft tissue disorders	Abdominal / retroperitoneal surgeries
Brain death	Bariatric surgeries
Cardiopulmonary arrest	Cardiac surgeries (except congenital heart diseases)
Cardiovascular	Cardiac surgeries for congenital heart diseases
Circulatory shock (excluding sepsis)	Colon, rectum and anal surgeries
Collagenosis, allergic, inflammatory and autoimmune disorders	Combined cardiac surgeries
Congenital heart diseases	Ear, nose and throat surgeries
Ear, nose and throat disorders	Endocrine surgeries
Endocrine/metabolic	Endovascular surgeries/procedures
Eye disorders	Esophageal surgeries
Female genital disorders	Eye surgeries
Gastrointestinal	Fetal surgeries
Hematological	Gastric surgeries
Hernias and abdominal wall defects	Gynecological and breast surgeries
Infection/sepsis	Head and neck surgeries
Liver, billiary tract, spleen and pancreas	Hernioplasties and abdominal wall defect surgical corrections
Male genital disorders	Invasive cardiac and endovascular procedures
Maternal or gestational related complications	Invasive procedures (except cardiovascular)
Monitoring	Liver, biliary tract and pancreatic surgeries
Multiple organ dysfunction	Lung, trachea and bronchi surgeries
Neurological/psychiatric	Neurosurgeries
Obstetrics	Obstetric procedures
Other/miscellaneous	Orthopedic surgeries
Palliative care	Other surgeries
Perinatal complications	Prostate surgeries
Potential organ and tissue donor	Skin and soft tissue surgeries
Preterm birth (prematurity)	Small bowel surgeries
Renal	Solid organ transplant
Respiratory (excluding sepsis/infection)	Spine surgeries
Solid organ transplant recipient	Surgical complications (peri/postoperative)
Trauma, nonsurgical	Surgical polytrauma
Tumors and oncological complications	Thoracic surgeries, other
	Urinary tract surgeries
	Vascular surgeries

Table 3S - Scoring systems available in the Epimed Monitor database

Severity of illness scores	SAPS 3,[1] EPM Mortality, SAPS II*[2] and APACHE II*[3]
Comorbidities	Charlson Comorbidity Index[(4)12% (181]
Frailty	Modified Frailty Index[5]
Nurse workload	Nursing Activities Score*[6]
Organ dysfunction	SOFA*[7]
ICU LOS	EPM length of stay* and EPM prolonged LOS*
ICU readmission	EPM ICU readmission*

SAPS - Simplified Acute Physiology Score; EPM - Epimed Prediction Models; APACHE - Acute Physiology and Chronic Health Evaluation; ICU - intensive care unit; LOS - length of stay. * Optional.

Table 4S - Published articles using data totally or partially retrieved from Epimed Monitor Adult ICU System identified in a non-systematic search in PubMed

Title	Year	Multicenter	Countries	Patients (n)	Topic	Link
Delirium epidemiology in critical care (DECCA): an international study	2010	Yes	11 countries	975	*Delirium*	https://pubmed.ncbi.nlm.nih.gov/21092264/
Clinical outcomes of patients requiring ventilatory support in Brazilian intensive care units: a multicenter, prospective, cohort study	2013	Yes	Brazil	773	Respiratory	https://pubmed.ncbi.nlm.nih.gov/23557378/
The impact of performance status and comorbidities on the short-term prognosis of very elderly patients admitted to the ICU	2014	No	Brazil	1,129	ICU case-mix and outcomes	https://pubmed.ncbi.nlm.nih.gov/25071415/
Admission factors associated with prolonged (> 14 days) intensive care unit stay	2014	No	Brazil	3,257	ICU case-mix and outcomes	https://pubmed.ncbi.nlm.nih.gov/24268622/
Intensive care in patients with lung cancer: a multinational study	2014	Yes	Six countries	449	Cancer	https://pubmed.ncbi.nlm.nih.gov/24950981/
A gradient-boosted model analysis of the impact of body mass index on the short-term outcomes of critically ill medical patients	2015	No	Brazil	1,943	ICU case-mix and outcomes	https://pubmed.ncbi.nlm.nih.gov/26340154/
Organizational characteristics, outcomes, and resource use in 78 Brazilian intensive care units: the ORCHESTRA study	2015	Yes	Brazil	59,693	ICU organization and performance	https://pubmed.ncbi.nlm.nih.gov/26499477/
Septic shock: a major cause of hospital death after intensive care unit discharge	2015	No	Brazil	581	Infection / Sepsis	https://pubmed.ncbi.nlm.nih.gov/25909313/
Effects of organizational characteristics on outcomes and resource use in patients with cancer admitted to intensive care units	2016	Yes	Brazil	9,946	Cancer	https://ascopubs.org/doi/10.1200/JCO.2016.66.9549
Factors associated with mortality in severe community-acquired pneumonia: A multicenter cohort study	2017	Yes	Brazil	7,902	Infection / Sepsis	https://pubmed.ncbi.nlm.nih.gov/30502687/
Family care, visiting policies, ICU performance, and efficiency in resource use: insights from the ORCHESTRA study	2017	Yes	Brazil	59,693	ICU organization and performance	https://pubmed.ncbi.nlm.nih.gov/28028553/
The Epimed Monitor ICU Database®: a cloud-based national registry for adult intensive care unit patients in Brazil	2017	Yes	Brazil	> 1,300,000	ICU organization and performance	https://pubmed.ncbi.nlm.nih.gov/29211187/
External validation of SAPS 3 and MPM0-III scores in 48,816 patients from 72 Brazilian ICUs	2017	Yes	Brazil	48,816	ICU organization and performance	https://pubmed.ncbi.nlm.nih.gov/28523584/
The effects of performance status one week before hospital admission on the outcomes of critically ill patients	2017	Yes	Brazil	59,693	ICU case-mix and outcomes	https://pubmed.ncbi.nlm.nih.gov/27686352/
Characteristics and outcome of patients with newly diagnosed advanced or metastatic lung cancer admitted to intensive care units (ICUs)	2018	Yes	Six countries	100	Cancer	https://pubmed.ncbi.nlm.nih.gov/30076547/
A comparison of mortality from sepsis in Brazil and England: the impact of heterogeneity in general and sepsis-specific patient characteristics	2018	Yes	Brazil/UK	34,150	Infection / Sepsis	https://pubmed.ncbi.nlm.nih.gov/30247269/

Title	Year	Multicenter	Countries	Patients (n)	Topic	Link
Role of organizational factors on the 'weekend effect' in critically ill patients in Brazil: a retrospective cohort analysis	2018	Yes	Brazil	59,614	ICU organization and performance	https://pubmed.ncbi.nlm.nih.gov/29371274/
Association of frailty with short-term outcomes, organ support and resource use in critically ill patients	2018	Yes	Brazil	129,680	ICU case-mix and outcomes	https://pubmed.ncbi.nlm.nih.gov/30105600/
ICU staffing feature phenotypes and their relationship with patients' outcomes: an unsupervised machine learning analysis	2019	Yes	Brazil	129,680	ICU organization and performance	https://pubmed.ncbi.nlm.nih.gov/31595349/
Modulators of systemic inflammatory response syndrome presence in patients admitted to intensive care units with acute infection: a Bayesian network approach	2019	Yes	Brazil	14,548	SIRS	https://pubmed.ncbi.nlm.nih.gov/30868180/
Customization and external validation of the Simplified Mortality Score for the Intensive Care Unit (SMS-ICU) in Brazilian critically ill patients	2020	Yes	Brazil	356,382	ICU organization and performance	https://pubmed.ncbi.nlm.nih.gov/32585439/
Central nervous system infection in the intensive care unit: development and validation of a multi-parameter diagnostic prediction tool to identify suspected patients	2021	No	Brazil	783	Infection / Sepsis	https://pubmed.ncbi.nlm.nih.gov/34843551/
The association of the COVID-19 pandemic and short-term outcomes of non-COVID-19 critically ill patients: an observational cohort study in Brazilian ICUs	2021	Yes	Brazil	644,644	ICU organization and performance	https://pubmed.ncbi.nlm.nih.gov/34518905/
Clinical characteristics and in-hospital mortality of cardiac arrest survivors in Brazil: a large retrospective multicenter cohort study	2021	Yes	Brazil	2,296	Neurocritical care	https://pubmed.ncbi.nlm.nih.gov/34345824/
SAPS-3 performance for hospital mortality prediction in 30,571 patients with COVID-19 admitted to ICUs in Brazil	2021	Yes	Brazil	30,571	COVID-19	https://pubmed.ncbi.nlm.nih.gov/34244829/
Evolving changes in mortality of 13,301 critically ill adult patients with COVID-19 over 8 months	2021	Yes	Brazil	13,301	COVID-19	https://pubmed.ncbi.nlm.nih.gov/33852032/
Elderly patients with cancer admitted to intensive care unit: A multicenter study in a middle-income country	2021	Yes	Brazil	4,604	Cancer	https://pubmed.ncbi.nlm.nih.gov/32822433/
Trends in clinical profiles, organ support use and outcomes of patients with cancer requiring unplanned ICU admission: a multicenter cohort study	2021	Yes	Brazil	32,096	Cancer	https://pubmed.ncbi.nlm.nih.gov/32770267/
Validation of a new data-driven SLOSR ICU efficiency measure compared to the traditional SRU	2021	Yes	Brazil	997	Neurocritical care	https://pubmed.ncbi.nlm.nih.gov/33150574/
Characteristics and outcomes of autologous hematopoietic stem cell transplant recipients admitted to intensive care units: a multicenter study	2022	Yes	Brazil	301	Cancer	https://pubmed.ncbi.nlm.nih.gov/35636348/
Comparing continuous versus categorical measures to assess and benchmark intensive care unit performance	2022	Yes	Brazil / Uruguay	277,459	ICU organization and performance	https://pubmed.ncbi.nlm.nih.gov/35576635/
Leveraging a national cloud-based intensive care registry for COVID-19 surveillance, research and case-mix evaluation in Brazil	2022	Yes	Brazil	192,500	COVID-19	https://pubmed.ncbi.nlm.nih.gov/35946649/

Continue...

...continuation

Title	Year	Multicenter	Countries	Patients (n)	Topic	Link
Hospital length of stay and 30-day mortality prediction in stroke: a machine learning analysis of 17,000 ICU admissions in Brazil	2022	Yes	Brazil	17,000	Neurocritical care	https://pubmed.ncbi.nlm.nih.gov/34345824/
Trends in intensive care admissions and outcomes of stroke patients over 10 years in Brazil: impact of the COVID-19 pandemic	2022	Yes	Brazil	17,115	Neurocritical care	https://pubmed.ncbi.nlm.nih.gov/36347322/
IMPACTO-MR: a Brazilian nationwide platform study to assess infections and multidrug resistance in intensive care units	2022	Yes	Brazil	33,983	Infection / Sepsis	https://pubmed.ncbi.nlm.nih.gov/36888821/
Geoeconomic variations in epidemiology, ventilation management, and outcomes in invasively ventilated intensive care unit patients without acute respiratory distress syndrome: a pooled analysis of four observational studies	2022	Yes	54 countries	773	Respiratory	https://pubmed.ncbi.nlm.nih.gov/34914899/
The association between prepandemic ICU performance and mortality variation in COVID-19: a multicenter cohort study of 35,619 critically ill patients	2023	Yes	Brazil	35,619	ICU organization and performance	https://pubmed.ncbi.nlm.nih.gov/37838338/
Clinical characteristics and outcomes of patients with COVID-19 admitted to the intensive care unit during the first and second waves of the pandemic in Brazil: a single-center retrospective cohort study	2023	No	Brazil	1,427	COVID-19	https://pubmed.ncbi.nlm.nih.gov/37493832/
Profiling and benchmarking central nervous system infections in an infectious diseases intensive care unit	2023	No	Brazil	785	Infection / Sepsis	https://pubmed.ncbi.nlm.nih.gov/37455413/
Non-COVID-19 intensive care admissions during the pandemic: a multinational registry-based study	2023	Yes	15 countries	793,768	ICU case-mix and outcomes	https://pubmed.ncbi.nlm.nih.gov/37225417/
Variants of concern and clinical outcomes in critically ill COVID-19 patients	2023	Yes	Brazil	47,465	COVID-19	https://pubmed.ncbi.nlm.nih.gov/37067557/
Oxygen debt as predictor of mortality and multiple organ dysfunction syndrome in severe COVID-19 patients: a retrospective study	2024	No	Colombia	708	COVID-19	https://pubmed.ncbi.nlm.nih.gov/37876236/
Development and validation of a machine learning model to predict the use of renal replacement therapy in 14,374 patients with COVID-19	2024	Yes	Brazil	14,374	COVID-19	https://pubmed.ncbi.nlm.nih.gov/21092264/
Generalizing the application of machine learning predictive models across different populations: does a model to predict the use of renal replacement therapy in critically ill COVID-19 patients apply to general intensive care unit patients?	2024	Yes	Brazil	14,374	COVID-19	https://pubmed.ncbi.nlm.nih.gov/38656079/

* PubMed Search performed on April 29th, 2024.

REFERENCES

1. Moreno RP, Metnitz PG, Almeida E, Jordan B, Bauer P, Campos RA, Iapichino G, Edbrooke D, Capuzzo M, Le Gall JR; SAPS 3 Investigators. SAPS 3--From evaluation of the patient to evaluation of the intensive care unit. Part 2: Development of a prognostic model for hospital mortality at ICU admission. Intensive Care Med. 2005;31(10):1345-55
2. Le Gall JR, Lemeshow S, Saulnier F. A new Simplified Acute Physiology Score (SAPS II) based on a European/North American multicenter study. JAMA. 1993;270(24):2957-63.
3. Knaus WA, Draper EA, Wagner DP, Zimmerman JE. APACHE II: a severity of disease classification system. Crit Care Med. 1985;13(10):818-29.
4. Charlson ME, Pompei P, Ales KL, MacKenzie CR. A new method of classifying prognostic comorbidity in longitudinal studies: development and validation. J Chronic Dis. 1987;40(5):373-83.
5. Zampieri FG, Iwashyna TJ, Viglianti EM, Taniguchi LU, Viana WN, Costa R, Corrêa TD, Moreira CE, Maia MO, Moralez GM, Lisboa T, Ferez MA, Freitas CE, de Carvalho CB, Mazza BF, Lima MF, Ramos GV, Silva AR, Bozza FA, Salluh JI, Soares M; ORCHESTRA Study Investigators. Association of frailty with short-term outcomes, organ support and resource use in critically ill patients. Intensive Care Med. 2018;44(9):1512-20.
6. Miranda DR, Nap R, de Rijk A, Schaufeli W, Iapichino G; TISS Working Group. Therapeutic Intervention Scoring System. Nursing activities score. Crit Care Med. 2003;31(2):374-82.
7. Vincent JL, Moreno R, Takala J, Willatts S, De Mendonça A, Bruining H, et al. The SOFA (Sepsis-related Organ Failure Assessment) score to describe organ dysfunction/failure. On behalf of the Working Group on Sepsis-Related Problems of the European Society of Intensive Care Medicine. Intensive Care Med. 1996;22(7):707-10.
