# Supplementary material for: Update on the Epimed Monitor Adult ICU Database: 15 years of its use in national registries, quality improvement initiatives and clinical research
Source: Crit Care Sci. 2024 Aug 15;36:e20240150en. doi: 10.62675/2965-2774.20240150-en (PMC11463981; doi:10.62675/2965-2774.20240150-en)
Supplement: Translation

# Translation: Atualização da *Epimed Monitor Adult ICU Database* : 15 anos de uso em registros nacionais, iniciativas de melhoria da qualidade e pesquisa clínica

**DOI:** 10.62675/2965-2774.20240150-pt

**Authors:** Marcio Soares, Lunna Perdigão Borges, Leonardo dos Santos Lourenco Bastos, Fernando Godinho Zampieri, Gabriel Alves Miranda, Pedro Kurtz, Suzana Margareth Lobo, Lucas Rodrigo Garcia de Mello, Gastón Burghi, Ederlon Rezende, Otávio Tavares Ranzani, Jorge Ibrain Figueira Salluh

**Affiliations:** 1 Rio de Janeiro RJ Brasil Instituto D’Or de Pesquisa e Ensino - Rio de Janeiro (RJ), Brasil.; 2 Rio de Janeiro RJ Brasil Epimed Solutions - Rio de Janeiro (RJ), Brasil.; 3 Rio de Janeiro RJ Brasil Departamento de Engenharia Industrial, Pontifícia Universidade Católica do Rio de Janeiro - Rio de Janeiro (RJ), Brasil.; 4 Edmonton Canadá Faculty of Medicine and Dentistry, University of Alberta - Edmonton, Canadá.; 5 São José do Rio Preto SP Brasil Divisão de Cuidades Intensivos. Hospital de Base, Faculdade de Medicina de São José do Rio Preto - São José do Rio Preto (SP), Brasil.; 6 Montividéu Uruguai Unidade de Terapia Intensiva, Hospital Maciel - Montividéu, Uruguai.; 7 São Paulo SP Brasil Unidade de Terapia Intensiva, Hospital do Servidor Público Estadual "Francisco Morato de Oliveira" - São Paulo (SP), Brasil.; 8 Barcelona Espanha Barcelona Institute for Global Health (ISGlobal) - Barcelona, Espanha.

**Keywords:** Bases de dados factuais, Sistema de registros, Melhoria de qualidade, Mortalidade hospitalar, Cuidados críticos, Unidades de terapia intensiva

## Abstract

Nas últimas décadas, foram disponibilizados vários bancos de dados de pacientes em estado crítico em países de baixa, média e alta renda de todos os continentes. Esses bancos de dados também são fontes ricas de dados para a vigilância de doenças emergentes, avaliação de desempenho e análise comparativa de unidades de terapia intensiva, projetos de melhoria da qualidade e pesquisa clínica. O banco de dados Epimed Monitor completa 15 anos em 2024 e se tornou um dos maiores desses bancos de dados. Nos últimos anos, observaram-se a rápida expansão geográfica, o aumento no número de unidades de terapia intensiva e hospitais participantes e a inclusão de diversas novas variáveis e escores, permitindo uma caracterização mais completa dos pacientes para facilitar estudos clínicos multicêntricos. Em dezembro de 2023, o banco de dados era usado sistematicamente por 23.852 leitos em 1.723 unidades de terapia intensiva e 763 hospitais de dez países, totalizando mais de 5,6 milhões de internações. Além disso, as sociedades de terapia intensiva adotaram o sistema e seu banco de dados para criar registros nacionais e cooperações internacionais. Nesta revisão, apresentamos uma descrição atualizada do banco de dados; relatamos experiências de seu uso em cuidados intensivos para iniciativas de melhoria da qualidade, registros nacionais e pesquisa clínica; e exploramos outras possíveis perspectivas e futuros avanços.

## INTRODUÇÃO

Nas últimas décadas, o crescente interesse em pesquisa clínica, epidemiologia, melhoria da qualidade e avaliação de desempenho de unidades de terapia intensiva (UTI), aliado à adoção em expansão da informática na área da saúde, resultou na criação de grandes bancos de dados de pacientes em estado crítico em todo o mundo. Atualmente, alguns grandes pacotes de dados contemporâneos que compreendem dados granulares sobre internação em UTI, obtidos de prontuários médicos eletrônicos totalmente dedicados à pesquisa clínica, fornecem aos pesquisadores livre acesso aos dados.^( 1 )^Além disso, foram criados e implementados mundialmente grandes bancos de dados regionais e nacionais que reúnem dados de combinação de casos, uso de recursos e desfechos de pacientes consecutivos em UTI, chamados de registros de UTI.^( 2 )^Essas iniciativas, tanto em países de baixa como de média e alta renda de todos os continentes, são valiosas fontes de dados para a vigilância de doenças emergentes, avaliação e análise comparativa do desempenho da UTI, projetos de melhoria da qualidade e pesquisa clínica (desde estudos observacionais até ensaios integrados a ensaios clínicos randomizados [ECRs]).

Há 15 anos, foi criado o banco de dados Epimed Monitor, uma plataforma em nuvem de gestão de desempenho clínico e avaliação comparativa de UTIs.^( 3 )^Desde 2009, o banco de dados cresceu em número de UTIs e internações e se expandiu para outros países, além do Brasil, tornando-se um dos maiores bancos de dados de cuidados intensivos do mundo. O objetivo inicial do banco de dados era avaliar o desempenho das UTIs e monitorar as iniciativas de melhoria da qualidade por parte dos gestores de UTIs e hospitais. Em 2017, um estudo publicado nesta revista descreveu o potencial da plataforma e do conteúdo do banco de dados para a pesquisa em terapia intensiva no Brasil.^( 3 )^Ao longo dos anos, as sociedades de terapia intensiva também adotaram o sistema e seu banco de dados para instituir registros nacionais e cooperações internacionais, como o Registro Nacional de Terapias Intensivas (UTIs Brasileiras).^( 4 , 5 )^Além disso, o banco de dados foi incrementado com diversas novas variáveis e escores após avanços científicos, tanto na pesquisa técnica, quanto na clínica. Assim, a presente revisão tem como objetivos atualizar a descrição do banco de dados; relatar experiências de seu uso em cuidados críticos em registros nacionais, iniciativas de melhoria da qualidade e pesquisa clínica; e explorar outras possíveis perspectivas e avanços futuros. Embora existam versões do sistema para UTIs pediátricas e neonatais, aqui nos concentramos apenas no banco de dados de UTI adulto.

### Descrição do banco de dados

#### Participação no banco de dados Epimed Monitor

A participação no banco de dados Epimed Monitor em todos os países é voluntária e regulada por um contrato comercial com uma empresa de tecnologia da informação (Epimed Solutions^®^, Rio de Janeiro, Brasil), que é responsável pelo desenvolvimento, atualização, segurança, privacidade e proteção dos dados, pela cópia de segurança e pelo suporte a todos os usuários do sistema. A maioria das UTIs usa a versão completa do sistema, mas algumas usam uma versão padrão gratuita disponível nos registros nacionais. O sistema está disponível em holandês, inglês, francês, alemão, português e espanhol. As palavras e os termos podem ser adaptados para atender às especificidades culturais locais (por exemplo, português brasileiro ou lusitano), conforme necessário, mantendo os mesmos códigos internos.

#### Registro e processamento de dados e controle de qualidade

Os dados são registrados em um formulário eletrônico de notificação de casos (eCRF) estruturado e hierárquico, que possui um formato básico de dados obrigatórios, que combina a integração com os prontuários eletrônicos (médicos e/ou administrativos) do paciente (PEPs) e a entrada manual de dados, dependendo da infraestrutura de tecnologia da informação dos hospitais, a qual pode variar desde a entrada manual completa de dados até a integração total com os PEPs. Na maioria das UTIs, os dados administrativos (demográficos, de internação e de alta) e laboratoriais são integrados, e um gestor de casos dedicado (geralmente enfermeiros) é responsável por inserir os dados clínicos de cada paciente consecutivo no banco de dados. A empresa oferece treinamento inicial aos gestores de casos, seguido de programas educacionais contínuos e atualizações e retornos periódicos. Também ocorre treinamento online/ao vivo, com reuniões presenciais regulares (pelo menos bimestrais) com os usuários, nas quais, por exemplo, a qualidade e a integridade dos dados podem ser verificadas novamente com os usuários, conforme necessário. Em geral, as internações são registradas prospectivamente. Caso contrário, os prontuários são revisados, evitando a perda de dados, especialmente de pacientes internados na UTI nos fins de semana ou pacientes que morrem dentro de 24 horas após a internação. Para seções específicas do eCRF (por exemplo, dados relacionados a infecções, eventos adversos, *Nurse Activities Score* [NAS] e listas de verificação), outros membros da equipe multidisciplinar podem estar envolvidos na inclusão de dados. Em especial, são realizadas algumas personalizações para atender às regulamentações locais, conforme o país.

Atribui-se a cada internação um identificador sequencial exclusivo, que segue a ordem de todo o banco de dados, não de cada unidade, hospital ou país. No caso de reinternações, mesmo durante a mesma hospitalização, também é atribuído um novo número identificador exclusivo.

Embora não haja auditorias externas regulares, o banco de dados é estruturado para ter controles ativos que garantam a qualidade e a consistência dos dados. Não há campos de texto livre, e todas as variáveis são estruturadas com códigos internos vinculados. Para minimizar os erros de processamento, que abrangem as etapas de codificação e entrada de dados, as definições/rótulos de cada variável não só estão claramente indicadas no eCRF, como também estão disponíveis em uma planilha em PDF facilmente acessível na plataforma *on-line* . Para solucionar possíveis erros, o sistema fornece rotinas de validação interna durante o processo de entrada de dados ("verificação interativa"). Não são permitidos intervalos de datas inconsistentes. O preenchimento condicional também está presente para algumas variáveis específicas (por exemplo, diagnósticos, patógenos e antibióticos). Para dados fisiológicos e laboratoriais, os valores além do intervalo normal da variável são destacados para revisão, e valores implausíveis não podem ser registrados. Os coordenadores de UTI e os gestores de casos podem monitorar casos incompletos e avaliar a proporção de valores ausentes ou a incompletude de variáveis específicas durante um período. Além disso, as verificações *offline* podem ocorrer de forma aleatória, dependendo da demanda de cada unidade e para atualização e melhoria do banco de dados.

#### Estrutura do formulário eletrônico de notificação de casos

O eCRF é estruturado hierarquicamente em páginas de dados específicas para variáveis dependentes e independentes do tempo ( Tabela 1S - Material suplementar ). Cada ponto de dados inserido no banco de dados é seguido de uma data cronológica para manter o registro.

Os dados demográficos e administrativos incluem identificadores exclusivos, idade, sexo, se a reinternação ocorreu durante a mesma hospitalização (e se a reinternação ocorreu dentro de 24, 48 ou 72 horas após a alta da UTI), peso, altura, número do leito, origem da internação, datas e horários de internação e alta do hospital e da UTI e destino após a alta da UTI e do hospital. As comorbidades incluem todas as comorbidades do *Charlson Comorbidity Index* (CCI),^( 6 )^o *Simplified Acute Physiology Score 3* (SAPS 3),^( 7 )^o *Modified Frailty Index* (MFI),^( 8 , 9 )^o *Acute Physiology and Chronic Health Evaluation* (APACHE) II^( 10 )^e outras comorbidades que possam ser úteis à avaliação e à estratificação de risco em condições específicas (por exemplo, acidente vascular cerebral, doença arterial coronariana). Os dados referentes ao estado de saúde crônico na semana anterior à internação hospitalar foram adaptados da escala de desempenho do *Eastern Cooperative Oncology Group* (ECOG).^( 11 , 12 )^

As internações são classificadas como clínicas, cirurgia eletiva ou cirurgia de emergência/urgência com base na classificação do diagnóstico inicial. Uma lista de mais de 1.500 diagnósticos médicos e cirúrgicos predefinidos está disponível e organizada em vários domínios ( Tabela 2S - Material suplementar ). Os diagnósticos secundários na internação e durante a internação também podem ser registrados. Há uma codificação fundamentada na Classificação Internacional de Doenças - décima revisão (CID-10).

A tabela 1 mostra o suporte invasivo de órgãos e as complicações agudas na internação (± 1 hora) e dentro de 24 horas da internação na UTI consideradas no banco de dados. Os dados laboratoriais e fisiológicos na internação e durante as primeiras 24 horas também são coletados, representando os dados necessários para calcular adequadamente os escores de gravidade da doença. O suporte e as intervenções invasivas durante a internação na UTI incluem monitoramento e intervenções invasivas (monitoramento hemodinâmico minimamente invasivo, cateteres venosos centrais [CVC], vesicais, *Swan-Ganz* e arteriais), suporte e monitoramento neurológicos (pressão intracraniana, monitoramento da pressão de oxigênio tecidual, saturação de oxigênio venoso jugular, microdiálise cerebral e drenagem ventricular externa), suporte ventilatório (ventilação não invasiva, ventilação mecânica [VM], duração da VM, traqueostomia, cânula nasal de alto fluxo e óxido nítrico inalado), suporte cardiovascular (marcapasso transvenoso, cateter de bomba de balão intra-aórtico, drogas vasoativas e oxigenação por membrana extracorpórea), terapia de substituição renal (TSR), transfusões de sangue, agentes trombolíticos, nutrição parenteral, hipotermia terapêutica e plasmaférese.

As escalas de priorização da UTI recomendadas pela *Society of Critical Care Medicine* (SCCM)^( 13 )^e pelo Conselho Federal de Medicina (CFM)/Associação de Medicina Intensiva Brasileira (AMIB), por meio da resolução 2.156/2016 do CFM,^( 14 )^e as decisões de priorização de cuidados paliativos e decisões de fim de vida foram atualizadas a partir da última descrição do banco de dados.

Várias UTIs também registram *checklists* diários para adesão às melhores práticas de evidência (sedação, cuidados com dispositivos invasivos, VM, prevenção de pressão em úlceras, pacotes de sepse e pacotes para prevenção de infecções hospitalares).

#### Sistemas de escore

Há vários escores no banco de dados para diferentes finalidades e domínios ( Tabela 3S - Material suplementar ). Os escores calculados com base nos dados obrigatórios são SAPS 3, CCI e MFI, conforme descrito. O *Sequential Organ Failure Assessment* (SOFA) também pode ser estimada no primeiro dia da UTI e diariamente em páginas de dados dinâmicas ou aplicativos móveis. Várias UTIs avaliam a carga de trabalho do enfermeiro por meio do NAS.^( 15 )^Além disso, algumas UTIs também usam o APACHE II e o SAPS 2.

Recentemente, a Epimed Solutions^®^desenvolveu uma série de escores proprietários, o *Epimed Prediction Model* (EPM), que usa modelagem de aprendizado de máquina em conjuntos de dados contemporâneos de mais de 1 milhão de pacientes. Há EPMs para estimar o risco de mortalidade hospitalar, o tempo de internação na UTI, o risco de internação prolongada (superior ao 90º percentil do tempo de internação de cada diagnóstico) e o risco de reinternação na UTI dentro de 48 horas após a alta. O desempenho de todos os escores é reavaliado periodicamente para verificar a necessidade de atualizações, conforme o caso.^( 16 )^

#### Indicadores de qualidade e avaliação de desempenho

O monitoramento dos indicadores de qualidade e as avaliações de desempenho e eficiência da UTI são os principais objetivos do uso do sistema pelas UTIs e hospitais. Os principais indicadores de qualidade são aqueles recomendados pelas sociedades nacionais locais, incluindo, no Brasil, a Agência Nacional de Vigilância Sanitária (Anvisa),^( 17 , 18 )^e a força-tarefa da *European Society of Intensive Care Medicine* (ESICM).^( 19 )^Os indicadores de qualidade do banco de dados incluem taxas de mortalidade na UTI e no hospital; taxa de mortalidade padronizada (TMP) usando escores de gravidade da doença; reinternações precoces não planejadas na UTI (dentro de 24, 48 e 72 horas após a alta); tempo de internação na UTI e no hospital; taxas de uso de dispositivos invasivos (VM, CVC e cateter vesical); taxas de incidência de infecções associadas à assistência à saúde (pneumonia associada ao ventilador, infecção da corrente sanguínea associada ao cateter venoso central e infecção do trato urinário associada ao cateter); e avaliações qualitativas e quantitativas da carga de trabalho do enfermeiro e da adesão a pacotes para prevenir infecções associadas à assistência à saúde. O banco de dados Epimed Monitor fornece vigilância de incidentes e eventos adversos, como incidentes e complicações relacionados a transfusões, eventos adversos induzidos por medicamentos, extubação não intencional e úlceras por pressão, entre outras situações.

Além da TMP usada para avaliar o desempenho clínico, duas outras medidas são fornecidas para medir a eficiência do uso de recursos. A razão de utilização de recursos padronizada (TURP) estima a razão média de recursos observada em relação à esperada (a partir do tempo de internação na UTI) usada por paciente sobrevivente em uma UTI específica ajustada à mortalidade de acordo com o SAPS 3 ou o EPM.^( 20 )^A eficiência da UTI pode ser analisada combinando-se a TMP e a TURP em uma matriz de eficiência.^( 21 , 22 )^O sistema também fornece o taxa de duração da internação padronizada (TDIP), que é a razão entre o tempo de internação observado e o tempo de internação previsto na UTI, a partir do tempo de internação pelo EPM.

### Unidades de terapia intensiva participantes e características dos pacientes

#### Distribuição geográfica e ampliação do banco de dados

Em dezembro de 2023, o Sistema Epimed Monitor para UTI Adulto era usado regularmente em 23.852 leitos de 1.723 UTIs e 763 hospitais de 10 países ( Figura 1 ). No Brasil, o sistema foi adotado por UTIs, que representam aproximadamente 50% dos leitos de UTI adulto em 25 dos 27 estados brasileiros.^( 4 )^As figuras 2A e 2B mostram o crescimento contínuo do banco de dados desde 2010. Em 2020 e 2021, houve um aumento acentuado no número de leitos e UTIs necessários para atender às demandas da doença pelo coronavírus 2019 (COVID-19), com diminuição subsequente à medida que a pandemia foi progressivamente controlada ( Figura 2A ). O número de internações em UTIs também aumentou progressivamente, ultrapassando mais de 5,6 milhões no total, e espera-se que ultrapasse 1 milhão de internações por ano em 2024 ( Figura 2B ).

**Figura 1:** Hospitais e unidades de terapia intensiva em países que usam o banco de dados Epimed Monitor de unidade de terapia intensiva adulto com os respectivos anos iniciais.

UTI - unidade de terapia intensiva.

**Figura 2:** Tendências anuais (2010 - 2023) em (A) número de hospitais, unidades de terapia intensiva e leitos de unidades de terapia intensiva e (B) número total de internações em unidades de terapia intensiva.

UTI - unidade de terapia intensiva.

#### Diagnóstico de internação, uso de suporte de órgãos e desfechos

As figuras 3A e 3B ilustram o perfil dos diagnósticos de internação na UTI. Em média, dois terços foram internações clínicas, as quais, como esperado, aumentaram durante a pandemia da COVID-19, paralelamente a uma diminuição na proporção de pacientes cirúrgicos agendados. A figura 3B mostra as cinco categorias de diagnóstico mais frequentes de acordo com o tipo de internação.

**Figura 3:** Tendências anuais (2010 - 2023) em (A) proporção de pacientes conforme o tipo de internação e (B) as cinco categorias de diagnóstico mais frequentes em cada tipo de internação.

UTI - unidade de terapia intensiva.

As figuras 4 e 5 retratam as tendências do principal uso de suporte invasivo e os resultados ao longo do tempo, respectivamente. Como esperado, os resultados de 2020 a 2022 foram afetados pelo número desproporcional de pacientes graves com COVID-19 internados em UTIs durante esses anos. No entanto, em 2023, esses dados foram novamente mais comparáveis aos achados pré-pandêmicos. As intervenções de suporte a órgãos mais frequentes ao longo dos anos são mostradas na figura 4 . De 2010 a 2019, houve tendência de diminuição na proporção de pacientes que necessitaram de VM, a qual pode ser explicada, em parte, pelo uso mais amplo de métodos de ventilação não invasiva mais recentemente. Por outro lado, a frequência do uso de vasopressores e TSR permaneceu relativamente estável ao longo dos anos. O mesmo padrão de tendências de uso de suporte invasivo foi observado nos desfechos dos pacientes. O tempo de internação na UTI permaneceu estável, mas houve modesta redução no tempo de internação hospitalar durante o período pré-pandêmico ( Figuras 5A e 5B ). Da mesma forma, houve pequena redução na mortalidade hospitalar, que não foi explicada por alterações no SAPS 3 ( Figura 5C ). Além disso, durante a pandemia da COVID-19, o SAPS 3 não se alterou em relação aos anos não pandêmicos, reforçando a imprecisão de seu desempenho em pacientes com COVID-19.^( 23 )^

**Figura 4:** Tendências anuais (2010 - 2023) na frequência de ventilação mecânica, uso de vasopressores e terapia de substituição renal.

UTI - unidade de terapia intensiva; VM - ventilação mecânica; TSR - terapia de substituição renal.

**Figura 5:** Tendências anuais (2010 - 2023) em (A) unidade de terapia intensiva e (B) tempo de internação hospitalar ( *boxplot* ; os pontos azuis representam o valor médio); (C) *Simplified Acute Physiology Score 3* médio (com respectivos intervalos de confiança de 95%) e taxa de mortalidade hospitalar.

UTI - unidade de terapia intensiva; SAPS 3 - *Simplified Acute Physiology Score 3* ; DP - desvio-padrão.

### Registros nacionais que adotam o banco de dados

O UTIs Brasileiras foi o primeiro registro nacional apoiado pelo banco de dados Epimed Monitor da AMIB.^( 4 , 5 )^Com início em 2010, o UTIs Brasileiras agora abrange aproximadamente 50% de todos os leitos de UTI no Brasil. Ele foi seguido pelo registro uruguaio, em 2016,^( 24 )^e pelo belga, em 2017.^( 25 )^Até o fim de 2023, o banco de dados era usado em dez países, seis dos quais o adotaram no contexto de criação de registros nacionais ( Figura 1 ). Atualmente, com o apoio da Federação Pan-Americana e Ibérica de Medicina Crítica e Terapia Intensiva (FEPIMCTI), foi iniciado um projeto denominado UCIs Ibero-Americanas para engajar os países da região a iniciarem seus próprios registros nacionais compartilhando a mesma plataforma.^( 26 )^Além do Brasil e do Uruguai, os seguintes países concordaram em participar: México, Panamá, Colômbia, Equador, Chile, Peru, Paraguai e países representados pelo *Consejo Centroamericano y del Caribe de Terapia Intensiva* (COCECATI).

### Uso em iniciativas de melhoria da qualidade

Além das iniciativas em nível nacional por meio de registros de UTI, o sistema e o banco de dados também são usados para auxiliar e monitorar iniciativas de melhoria da qualidade em UTIs e hospitais. Uma grande rede de hospitais empregou o sistema para monitorar a implementação de medidas de processo associadas a auditorias e retorno para melhorar a assistência e os desfechos de pacientes com sepse e minimizar a sedação.^( 27 , 28 )^

### Pesquisa clínica

Em uma pesquisa não sistemática do banco de dados PubMed, identificamos 44 artigos sobre pacientes adultos em estado crítico cujos dados foram parcial ou totalmente obtidos do Epimed Monitor ( Tabela 4S - Material suplementar ). Havia 36 (82%) estudos multicêntricos. A maioria dos estudos incluiu apenas UTIs do Brasil (n = 36, 82%), e oito foram realizados em UTIs de outros países, incluindo quatro estudos multinacionais. O número médio de pacientes por estudo foi de 13.301 (variando de 100 a mais de 1.300.000). Os principais tópicos de interesse foram organização e desempenho na UTI (n = 10, 23%), COVID-19 (n = 8, 18%), combinação de casos e desfechos na UTI (n = 6, 14%), pacientes críticos com câncer (n = 6, 14%), infecção/sepse (n = 6, 14%) e cuidados neurocríticos (n = 4, 9%), entre outros. Notavelmente, o estudo ORCHESTRA ( *Organizational CHaractEriSTics in cRitical cAre* ), cujo objetivo é investigar as associações entre as características dos pacientes, os aspectos organizacionais da UTI, os desfechos dos pacientes e o desempenho e a eficiência nas UTIs, é o maior em número de publicações.^( 9 , 12 , 16 , 22 , 29 - 40 )^Esse estudo foi realizado em um período de 10 anos e inclui dados de mais de 600 mil pacientes e 250 UTIs do Brasil e do Uruguai.

### Perspectivas futuras

O crescimento do banco de dados Epimed Monitor de UTI adulto em termos de países, hospitais, UTIs e pacientes criará oportunidades para alavancar estudos colaborativos multicêntricos e multinacionais com dados padronizados, harmonizados e de alta qualidade. Além disso, pode reduzir o esforço e o ônus de realização de estudos clínicos, uma vez que a caracterização clínica abrangente dos pacientes, do uso de suporte e dos desfechos já foi rotineiramente coletada de forma prospectiva, como vem ocorrendo em outros países, como Austrália, Nova Zelândia e Reino Unido. Esse banco de dados pode permitir identificar centros com populações de pacientes-alvo para esses estudos; apoiar estimativas realistas do tamanho da amostra, do poder e do recrutamento para ensaios clínicos; e facilitar ensaios incorporados ao registro.^( 41 )^Os pesquisadores podem realizar estudos para validar e testar escores e medidas existentes usados regularmente em pacientes de cuidados intensivos e verificar a eventual necessidade de atualizações e personalizações locais por meio de dados contemporâneos.^( 16 , 23 , 36 )^No futuro, esse banco de dados poderá desempenhar um papel no rastreamento da evolução e da ocupação de leitos de UTI e fornecer informações quase em tempo real sobre os pacientes durante pandemias, como ocorreu com a COVID-19.^( 5 )^Por fim, várias novas perspectivas serão abertas com a disponibilidade de novos algoritmos de modelagem usando-se aprendizado de máquina e outras técnicas de inteligência artificial.

## CONCLUSÃO

O banco de dados Epimed Monitor de unidade de terapia intensiva adulto é um dos maiores bancos de dados de cuidados intensivos do mundo. Nos últimos anos, houve rápida expansão geográfica e aumento no número de unidades de terapia intensiva e hospitais participantes, com diversas novas variáveis e escores incluídos, permitindo uma caracterização mais completa dos pacientes para promover estudos clínicos multicêntricos. Um número cada vez maior de países está adotando bancos de dados para promover registros nacionais de unidades de terapia intensiva, monitorar o desempenho e a eficiência das unidades de terapia intensiva e apoiar iniciativas de melhoria da qualidade.

## MATERIAL SUPLEMENTAR
